# The Use of Spice Herbs May Reduce Chronic Inflammation and Improve the Quality of Life of Women with Metabolic Syndrome—A Narrative Review

**DOI:** 10.3390/nu18071018

**Published:** 2026-03-24

**Authors:** Anna Winiarska, Karolina Jachimowicz-Rogowska, Małgorzata Kwiecień, Ewa Stamirowska-Krzaczek, Klaudia Kałwa, Małgorzata Stryjecka, Agnieszka Tomczyk-Warunek, Piotr Olcha

**Affiliations:** 1 Institute of Animal Nutrition and Bromatology, University of Life Sciences in Lublin, Akademicka St. 13, 20-950 Lublin, Poland; karolina.jachimowicz@up.edu.pl (K.J.-R.); malgorzata.kwiecien@up.edu.pl (M.K.); 2 Institute of Human Nutrition and Agriculture, The University College of Applied Sciences in Chełm, Pocztowa St. 54, 22-100 Chełm, Poland; ekrzaczek@panschelm.edu.pl (E.S.-K.); kkalwa@panschelm.edu.pl (K.K.); mstryjecka@panschelm.edu.pl (M.S.); 3 Laboratory of Locomotor Systems Research, Department of Traumatology, Orthopedics and Rehabilitation, Medical University of Lublin, Jaczewskiego St. 8, 20-954 Lublin, Poland; agnieszka.tomczyk-warunek@umlub.edu.pl; 4 Department of Gynecology and Gynecological Endocrinology, Medical University of Lublin, Aleje Racławickie St. 23, 20-049 Lublin, Poland; piotr.olcha@umlub.pl

**Keywords:** metabolic syndrome, spice herbs, chronic inflammation, oxidative stress, bioactive compounds, estrogen-dependent neoplasms, women’s health

## Abstract

**Background:** Metabolic syndrome is a disorder characterised by the concomitant presence of obesity, hyperglycaemia, hypertension, hyperlipidaemia, and insulin resistance. An increasing body of research indicates that chronic inflammation, accompanied by oxidative stress and angiogenesis, plays a key role in the pathogenesis of the metabolic syndrome. Spice herbs may exert a beneficial effect when consumed daily in generally accepted amounts (1–3 g), thus providing relatively small quantities of bioactive compounds with anti-inflammatory properties. Their potential arises from regular long-term use rather than from the amount of bioactive substances delivered in a single dose. **Methods:** In this narrative review, we analysed data from the international literature on the effects of spice herbs (coriander, sage, mint, basil, rosemary, oregano and thyme) consumption on inflammation associated with metabolic syndrome in women. **Results:** The available literature provides limited data on the impact of spice herbs in the context of anti-inflammatory effects. A total of 124 publications were analysed, including 72 original research studies (48 involving humans) and 52 review articles and meta-analyses. Among the research articles included in the review, only 20 addressed both inflammation and at least one of the seven selected herbs: five were human studies, six involved laboratory animals, and eight were conducted in vitro. Analysis of the results from human studies demonstrated anti-inflammatory effects (decreases in TNF-α, IL-1β, IL-6, TLR4, hs-CRP) at daily doses not exceeding 3 g of individual herbs or 6.6 g of an herbal mixture. The use of spice herbs as a nutritional strategy to prevent chronic inflammation is supported by a growing body of scientific evidence. It should be emphasised that these studies are concerned with dietary support and prevention rather than with treatments that substitute for standard medical therapy. Incorporating spice herbs into the daily diet may represent a simple and safe approach to increasing the intake of anti-inflammatory bioactive compounds. **Conclusions:** Future research should focus on the precise determination of optimal doses and combinations of spice herbs to maximise benefits while avoiding potential adverse effects resulting from excessive intake of certain compounds or inappropriate selection of spice herbs. Long-term studies conducted in larger populations of women with metabolic syndrome are required, as physiological differences, particularly those related to oestrogens, may result in sex-specific effects. This review provides up-to-date information for further basic and clinical research on herbal medicine in metabolic syndrome.

## 1. Introduction

Metabolic syndrome is a disorder characterised by the concomitant presence of obesity, hyperglycaemia, hypertension, hyperlipidaemia, and insulin resistance [1]. These symptoms act synergistically to increase the risk of developing cardiovascular disease, stroke, and diabetes. Metabolic syndrome is also recognised as an important risk factor for various neoplasms, including oestrogen-dependent neoplasms [2]. Moreover, studies have shown that women with metabolic syndrome have poorer prognoses in cases of oestrogen-dependent neoplasms, and disease recurrence is more frequently observed in this population [2]. Metabolic syndrome is an increasing global public health concern. A comparison of global trends in the prevalence of women with metabolic syndrome revealed that it has approximately doubled since 2000 [3]. According to estimates, approximately 846 million women worldwide will be affected by metabolic syndrome in 2023 [3].

An increasing body of research indicates that chronic inflammation, accompanied by oxidative stress and angiogenesis, plays a key role in the pathogenesis of metabolic syndrome [2]. An effective approach to mitigating inflammation is the implementation of an anti-inflammatory diet based on unprocessed foods enriched with functional foods that are high in bioregulatory compounds [4]. This represents the most effective, cost-efficient, and straightforward strategy for the prevention and management of metabolic syndrome and its constituent disorders [1,5]. The management of metabolic syndrome also requires lifestyle modifications, including increased physical activity, smoking cessation, and reduction of salt and alcohol intake, alongside pharmacotherapy aimed at achieving weight loss [6]. The use of spice herbs is a simple and cost-effective approach to modifying the dietary habits of the population. They provide anti-inflammatory compounds [7] and enhance the flavour of meals, allowing for a reduction in the use of table salt [8]. Excessive salt intake is strongly associated with a range of non-communicable diseases, such as hypertension and obesity [9], which are components of the metabolic syndrome.

In this narrative review, we analysed data available in the international literature concerning the effects of spice herb consumption on inflammation associated with metabolic syndrome in women. A simple yet effective dietary intervention may improve the quality of life in women and constitute a key component of the prevention of diseases associated with metabolic syndrome, particularly oestrogen-dependent neoplasms.

## 2. Materials and Methods

A literature review was conducted in January–February 2026 using the Scopus, PubMed, Web of Science, and Google Scholar databases. In each database, the keywords “culinary herbs”, “metabolic syndrome”, “woman” and “inflammation” were entered in English. We assumed that only herbs consisting of the green aerial parts (leaves and stems, fresh and/or dried) and used as culinary spices would be included in the review, as these can be used daily and in a simple manner. After analysing the titles and synopses of research and review publications, it was concluded that the following spice herbs were most commonly referenced in the context of obesity due to their beneficial effects on the functioning of the gastrointestinal tract: coriander (*Coriandrum sativum* L.), sage (*Salvia officinalis* L.), mint (*Mentha* sp.), basil (*Ocimum basilicum* L.), rosemary (*Rosmarinus officinalis* L.), oregano (*Origanum vulgare* L.), and thyme (*Thymus vulgaris* L.). They are also found in herbal dietary supplements that support digestion and the proper functioning of the digestive tract. All of these herbs are used as culinary herbs and are also present in the Mediterranean diet, considered one of the healthiest diets worldwide.

Articles were included in the analysis if they:

(1) Contained information on the anti-obesogenic, antidiabetic, insulin-sensitising, blood pressure-lowering, and/or lipid-lowering effects of the previously selected spice herbs;
(2) Provided information on the use of herbs in metabolic syndrome in women;
(3) Supplied information on the mechanisms of anti-inflammatory action of bioactive components of the herbs;
(4) Were genuine studies (involving laboratory animals, humans, or in vitro), review articles, or clinical studies published in English;
(5) Were published from 2014 to 2025.

**Figure 1:**
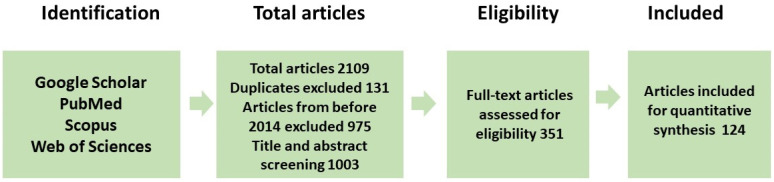
Methodology for reviewing the existing literature.

Papers unrelated to the subject matter were excluded, and the remaining texts were thoroughly analysed to select the most pertinent publications. The bibliographies of the analysed papers were consulted to identify any missing valuable articles. After removal of duplicates, a total of 124 publications were analysed (Figure 1), including 72 original research studies (48 involving humans) and 52 review articles and meta-analyses. Among the research articles included in the review, only 20 addressed both inflammation and at least one of the seven selected herbs: five were human studies, six involved laboratory animals, and eight were conducted in vitro. Two reviewers with clinical expertise in gynecology and dietetics independently evaluated the articles that met the inclusion criteria to determine which should be included in the review. Due to the narrative nature of this review, no formal meta-analytical procedure was applied; the results were synthesised qualitatively, emphasising the biological consistency of the observed effects.

## 3. Results

### 3.1. Inflammation in Metabolic Syndrome

Inflammation is the body’s physiological response to tissue damage or the presence of inflammatory stimuli. Metabolic syndrome is associated with inflammation, accompanied by elevated plasma concentrations of inflammatory biomarkers, such as high-sensitivity *C*-reactive protein (hs-CRP), interleukin-6 (IL-6), tumour necrosis factor-alpha (TNF-α), nuclear factor kappa B (NF-κB), interferon gamma (IFN-γ), and interleukin-1 beta (IL-1β) [10,11,12]. Inflammatory mediators play a key role in the initiation and regulation of the immune response, and their overproduction is strongly associated with metabolic and cardiovascular disorders [13].

Visceral adipose tissue is recognised as a key factor in the development of metabolic syndrome, serving as a metabolic trigger that activates multiple harmful pathways [13]. Chronic inflammation is a hallmark of both cellular and humoral immune responses within adipose tissue, with macrophages playing a central role [12]. Evidence indicates that inflammation in adipose tissue is critical for the onset of metabolic syndrome and its related complications, as it represents a convergence point for numerous molecular pathways that interact with the immune system and modulate organ function through cytokine signalling [12]. In metabolic syndrome, white adipose tissue produces increased amounts of inflammation-regulating molecules, primarily leptin, adiponectin, adropin, and visfatin, which influence both adipose tissue physiology and the functions of other organs [14]. Adipose tissue-derived mediators involved in energy metabolism also play a significant role in modulating the immune responses of the body. It has been demonstrated that leptin and adiponectin levels are directly associated with body mass index (BMI) and metabolic syndrome [15]. Moreover, the leptin-to-adiponectin ratio and low-grade inflammation play roles in the pathogenesis of type 2 diabetes and cardiovascular diseases [16]. Leptin and adiponectin exert opposing effects on low-grade inflammation: leptin increases the secretion of pro-inflammatory cytokines (e.g., TNF-α, IL-6), whereas adiponectin reduces the expression and release of pro-inflammatory mediators [16]. A strong positive correlation between adiponectin levels and insulin sensitivity has been reported [17]. In contrast, leptin does not directly stimulate monocytes to produce cytokines but induces sustained monocyte hyperresponsiveness, as demonstrated in an in vitro study [18]. Adropin and visfatin are also implicated in immune and inflammatory processes associated with metabolic syndrome. Adropin mitigates inflammation by downregulating the mRNA expression of pro-inflammatory cytokines, particularly IL-6 and TNF-α, and modulates the expression of peroxisome proliferator-activated receptor gamma (PPAR-γ) via activation of the intracellular AKT signalling pathway. This mechanism inhibits the differentiation of 3T3-L1 preadipocytes into mature adipocytes, thereby reducing fat accumulation [19]. Visfatin, a pro-inflammatory adipocytokine, promotes the production of pro-inflammatory cytokines, including IL-1, IL-6, IL-8, and TNF-α, by monocytes, and also induces chemotaxis of activated mononuclear cells [20,21]. Elevated visfatin levels are commonly observed under inflammatory conditions. In animal studies, visfatin has been shown to modulate the inflammatory response by regulating levels of pro-inflammatory eosinophils, myeloperoxidase, and cytokines [21].

Adipocytes modulate the inflammatory response by secreting signalling molecules with both pro- and anti-inflammatory effects. Free fatty acids directly stimulate Toll-like receptor 4 (TLR4), promoting an inflammatory cascade [12].

In turn, tenascin C, an endogenous activator of TLR4, exhibits increased levels in visceral adipose tissue in obese individuals, and the expression of anti-inflammatory TLR9 is also increased in obese individuals [22,23]. The NF-κB signalling pathway plays a significant role in local and systemic inflammation induced by obesity [24]. Its activation stimulates the expression of kinases that amplify the inflammatory processes. Reactive oxygen species (ROS) induce NF-κB-dependent apoptosis, accompanied by inflammation resulting from increased production of pro-inflammatory adipokines. Dead adipocytes and macrophages form crown-like structures that are common in visceral fat depots. These macrophages stimulate the release of free fatty acids from adipocytes via TNF-α, which bind to TLR4 on adipocytes and macrophages, triggering the activation of NF-κB signalling and the release of IL-1β by macrophages [12]. The macrophage population in adipose tissue consists of two main categories: M1 macrophages, which exhibit pro-inflammatory characteristics and predominate in obese individuals, and M2 macrophages, which exhibit anti-inflammatory activity and predominate in lean individuals [12].

### 3.2. Metabolic Syndrome in Women

Sex-related differences in inflammation associated with metabolic syndrome have been identified in women and men [18,25]. In women, anti-inflammatory adiponectin levels are typically reduced, whereas in men, excessive production of pro-inflammatory markers, particularly IL-6 and leptin, is observed. It has been demonstrated that, in women with metabolic syndrome, waist circumference, BMI, Homeostatic Model Assessment (HOMA) index, glucose levels, total cholesterol, and triglycerides are significantly elevated, concomitant with overexpression of leptin and nerve growth factor (NGF) in plasma and reduced adiponectin levels [14]. Notably, in the cited study, the immunohistochemical expression of leptin and NGF was strong. Excess body weight is a primary risk factor for metabolic syndromes [13]. However, a high prevalence of metabolic syndrome risk factors has been reported even in individuals with BMI within the upper normal range (n = 13,172, age 37–66 years), highlighting the need for effective diagnosis of metabolic obesity in individuals with normal body weight [26].

Sex hormones regulate lipolysis and lipogenesis, modulate the expression of transcription factors, influence adipocyte proliferation, and control the production of adipokines, including resistin, adiponectin, leptin, and angiotensin [27]. Estrogen deficiency exacerbates adipose tissue dysfunction by suppressing oestrogen receptor α signalling, increasing oestrogen receptor β activity, and impairing mitochondrial function, insulin sensitivity, and lipid oxidation [28]. Changes in hormone levels during menopause modulate fat metabolism and alter fat distribution, favouring visceral fat accumulation, independent of lifestyle-related factors. Because visceral fat is considered a primary metabolic trigger for inflammation, excess body weight in perimenopausal women is associated with elevated levels of IL-6, *C*-reactive protein (CRP), and insulin resistance, as well as BMI-dependent correlations between chemokines and the Systemic Immune-Inflammation Index (SII) and Systemic Inflammatory Response Index (SIRI) [13]. These factors contribute to an increased prevalence of metabolic syndrome during menopause as a result of estrogen deficiency; menopausal age is therefore regarded both as a risk factor for metabolic syndrome and for each of its individual components.

Metabolic syndrome increases the risk of various neoplasms, including oestrogen-dependent tumours, which are associated with poorer prognoses [2]. These include endometrial, breast, and cervical cancers, as well as endometriosis [2,29,30,31]. Physiological markers of these conditions include dysfunction of the antioxidant system and progressive inflammation [2].

### 3.3. Dietary Influence on Inflammation

Bioactive dietary components affect the onset and progression of inflammation, exerting both positive and negative influences. A study of 6538 individuals revealed a significant relationship between a diet rich in pro-inflammatory components, assessed using the Dietary Inflammatory Index (DII), and the risk of developing metabolic syndrome and its components [10]. Comparable results were obtained by Nikniaz et al. [32] in a study of 606 individuals with metabolic syndrome. Delgado-Alarcón et al. [33] suggested that the type of dietary fat influences pro-inflammatory cytokine production. In a three-month intervention involving 60 women, the effects of breakfasts enriched with different fatty acids were assessed: polyunsaturated fatty acids (PUFA–margarine), monounsaturated fatty acids (MUFA–extra virgin olive oil), or saturated fatty acids (SFA–butter) on inflammatory markers. The most pronounced effects were observed in the MUFA group, in which a statistically significant reduction in plasma IL-6 and vascular endothelial growth factor (VEGF) was noted. Breakfast enriched with PUFA was also beneficial, inducing a significant decrease in epidermal growth factor (EGF) levels. In contrast, no significant influence on the inflammatory markers was observed in the SFA group. These findings indicate that modifying the type of dietary fatty acids, particularly by increasing MUFA or PUFA intake, may be advantageous in reducing circulating inflammatory markers.

In individuals with metabolic inflammation, regular consumption of dietary fibre reduces the levels of inflammatory markers. This was demonstrated in a study by Hall et al. [34], in which 60 individuals with metabolic syndrome were assigned to receive a daily 10 g mixed prebiotic supplement alongside healthy eating advice (*n* = 40), while the control group received dietary advice only (*n* = 20). The intervention group exhibited a significant reduction in hs-CRP levels compared to the control group. Furthermore, after 12 weeks, an increase in faecal *Bifidobacterium* and *Parabacteroides* abundance was observed in the intervention group; these bacteria are recognised producers of short-chain fatty acids (SCFAs), a mechanism contributing to reduced gut and systemic inflammation. Comparable results were reported by the same authors in a subsequent study [35], in which 66 individuals with prediabetes receiving 20 g of a fibre mixture daily showed reduced CRP levels and improved insulin resistance markers (HbA1c, insulin sensitivity). Reducing inflammation and improving metabolic parameters (e.g., insulin resistance and lipid profile) are crucial in the context of metabolic syndrome.

Data from 33 randomised controlled trials suggest that adherence to the Mediterranean diet can significantly lower selected inflammatory markers compared with a standard diet [36]. The Mediterranean diet is rich in vegetables and fruits (containing polyphenols and other antioxidants), whole grains, olive oil (MUFA), fish (omega-3 PUFA), nuts and seeds. It contains a few sources of saturated fatty acids and abundant plant-based foods [37]. The recommended intake includes a higher consumption of MUFA, vegetables, fruits, legumes, nuts, and fish; moderate consumption of dairy products, meat, and wine; and a limited intake of SFA [38]. The beneficial impact of the Mediterranean diet on metabolic status and related inflammatory processes was demonstrated in a study of 70 girls with metabolic syndrome. After 12 weeks of adherence to dietary recommendations, the intervention group exhibited reductions in body weight, BMI, and waist circumference, as well as improvements in inflammatory markers (IL-6 and hs-CRP) and metabolic parameters (blood pressure, fasting glucose, triglycerides, low-density lipoprotein cholesterol, high-density lipoprotein cholesterol, and HOMA-IR) compared with the control group [39]. In a study by Duś-Żuchowska et al. [40], involving 144 postmenopausal women at risk of metabolic syndrome, 16 weeks of adherence to the Mediterranean diet resulted in a significant decrease in hs-CRP levels relative to baseline. Polyphenol-rich foods, particularly anthocyanin- and flavonoid-rich blueberries, may help reduce inflammation. In a group of 15 adults with metabolic syndrome consuming 45 g of freeze-dried blueberries daily in a yoghurt smoothie for six weeks, expression of pro-inflammatory genes (TNF-α, IL-6, TLR4) in monocytes was significantly reduced, and circulating granulocyte–macrophage colony-stimulating factor (GM-CSF) levels decreased compared with a placebo group (*n* = 12), indicating the potential for anti-inflammatory immunometabolic modulation [41].

### 3.4. Spice Herbs with Anti-Inflammatory Properties

Basil (*Ocimum basilicum* L.) is a source of aromatic compounds and essential oils that contain biologically active constituents. Linalool is the principal constituent of basil essential oil (28.6–60.6%), followed by estragole, methyl cinnamate, epi-α-cadinol, α-bergamotene, γ-cadinene (3.3–5.4%), germacrene D (1.1–3.3%), and camphor (1.1–3.1%). Other compounds identified in basil leaves include myrcene, pinene (0.1%), terpineol (0.7 ± 0.0–1.0 ± 0.0%), 1,8-cineole (0.2 ± 0.0–1.2 ± 0.0%), eugenol (4.5%), and methyleugenol (39.3%) [42,43].

The active constituents of spearmint (*Mentha spicata*) include mint essential oil, flavonoids (diosmin and diosmetin), phenolic acids, and lignans. The most abundant compound in mint oil is carvone (68.4%), which gives spearmint its characteristic aroma. Mint essential oil also contains substantial amounts of limonene (14.8%), dihydrocarvone (7.2%), and 1,8-cineole (4.5%) [44].

Sage (*Salvia officinalis* L.) contains primary anti-inflammatory compounds such as rosmarinic acid (9.731 ± 0.067 mg/g, 0.97%), carnosol (5.541 ± 0.276 mg/g, 0.55%), and carnosic acid (2.915 ± 0.248 mg/g, 0.3%). Its main essential oil constituents include R-thujone (11.55–19.23%), viridiflorol (9.94–19.46%), 1,8-cineole (8.85–15.60%), camphor (5.08–15.06%), manool (5.52–13.06%), β-caryophyllene (2.63–9.24%), R-humulene (1.93–8.94%), and β-thujone (5.45–6.17%) [45,46].

Essential oils derived from thyme (*Thymus vulgaris* L.) contain 20–58% thymol and 15–28% *p*-cymene, followed by γ-terpinene (4–10%), linalool (0.7–6.5%), carvacrol (1–4%), myrcene (1–3%), 1,8-cineole (0.8%), and borneol (0.7–1.7%) [47].

The compounds contributing to oregano (*Origanum vulgare*) flavour include thymol (39.3%), caryophyllene (17.2%), pinene (10.5%), carvacrol (7.6%), limonene, and ocimene [48]. Oregano also contains polyphenols, including caffeic, *p*-coumaric, and rosmarinic acids, which exhibit antioxidant activity and prevent lipid peroxidation [49].

Coriander (*Coriandrum sativum* L.) contains a range of polyphenols whose antioxidant properties are derived from the chemical characteristics of phenolic acids and flavonoids [50]. In dried coriander leaves, one important flavonoid, quercetin, was present at approximately 18.8 mg/100 g dry weight (0.02%) [51]. The leaves contain not only quercetin but also predominantly its glycosidic derivatives, which may constitute a considerably larger proportion of polyphenols in the raw material. Flavonoid glycosides (e.g., rutinose and glucuronide) are chemically bound to sugars and represent the primary storage form of flavonoids in many plants. The most abundant compounds are quercetin-3-O-rutinoside (ca. 448 mg/100 g; 0.45%), quercetin-3-O-glucuronide (ca. 105 mg/100 g; 0.11%), and quercetin-3-O-glucoside (ca. 38 mg/100 g; 0.04%) [52].

Rosemary (*Rosmarinus officinalis* L.) contains multiple phytochemicals, including rosmarinic acid (33.5 mg/g, 3.35%), camphor, caffeic acid, ursolic acid, betulinic acid, carnosic acid (3.8–4.6%), and carnosol (0.1–0.5%) [53,54]. The principal essential oil constituents of rosemary are borneol (26.5%), α-terpinene (15.6%), and α-pinene (12.7%) [55].

**Figure 2:**
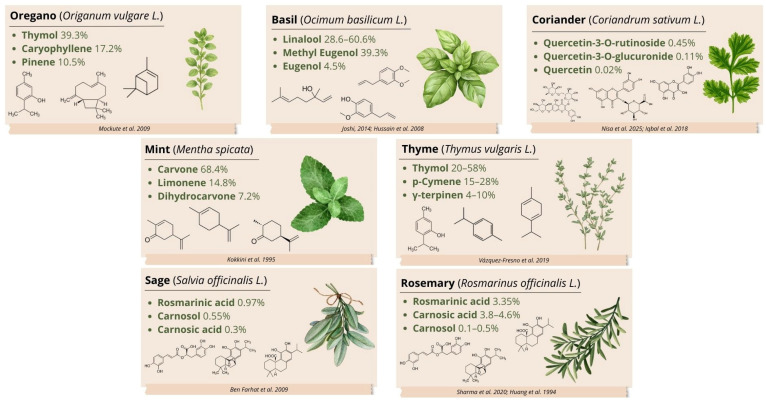
Anti-inflammatory bioactive compounds in spice herbs in the context of metabolic syndrome [42,43,44,46,47,48,51,52,53,54].

The anti-inflammatory bioactive compounds in spice herbs are shown in Figure 2.

The anti-inflammatory effects of herbs are primarily due to their polyphenolic compounds. Flavonoids, especially flavonols, can activate lipoxygenase and weaken adipogenesis by regulating the AMP-activated protein kinase (AMPK) pathway. AMPK activation is triggered by an increase in AMP concentration during periods of energy deficiency, resulting in the activation of catabolic pathways and inhibition of energy consumption processes [56,57]. Flavonoids most likely modulate the number of cellular signal pathways affecting carbohydrate digestion, fat accumulation, insulin release rate, and glucose uptake in insulin-sensitive tissues [56]. Studies have shown that polyphenols regulate metabolism in adipocytes to inhibit the growth of fatty tissues and reduce their vitality and differentiation [56,58]. Phenolic, chlorogenic, and coumaric acids effectively inhibited the growth of preadipocyte cells in mice [59,60]. They can also reduce the biosynthesis of fatty acids and accumulation of triglycerides, stimulate lipolysis and β-oxidation of fatty acids, and reduce inflammation by inhibiting the expression of strong pro-inflammatory TNF-α adipokines, monocyte chemotactic protein-1 (MCP-1), and type 1 plasminogen activator inhibitor (PAI-1), as well as increase the production of anti-inflammatory adiponectin in adipocytes [61,62]. Polyphenols modulate signal pathways, including AMPK, the receptor activated by γ peroxisome proliferators, and NF-κB–regulating adipogenesis, antioxidative and anti-inflammatory response, and the expression of some adipokine genes reacting to oxidation, including TNF-α [58,63]. Saponins exhibit anti-inflammatory, anti-lipidemic (by inhibiting pancreatic lipase), hypocholesterolemic, and hypoglycaemic properties [64]. In studies conducted on rats, saponins increased the phosphorylation of AMPK in a dose-dependent manner, suggesting their direct regulatory role in the activation of AMPK in adipocytes [65]. Saponins reduce the accumulation of internal fat by inhibiting adipogenic transcription factors responsible for AMPK signalling, such as CCAAT/enhancer-binding protein alpha (C/EBPα) and PPARγ2 [64]. Alkaloids increase energy expenditure, reduce appetite, and inhibit adipocyte differentiation and pancreatic lipase secretion. Most alkaloids are α-adrenergic antagonists, although some exhibit β-adrenergic antagonist properties [56].

### 3.5. Gut Microbiota–Inflammation–Polyphenols Interplay

A properly structured gut microbiota is essential for optimal functioning of the body. Dysbiosis is associated with the development of pathological conditions, including chronic diseases such as metabolic syndromes [66]. Inflammation can be triggered by the structural components of bacteria, which activate cascades of inflammatory pathways involving interleukins and other cytokines. Conversely, bacterial metabolites, primarily SCFAs, enhance intestinal barrier integrity and suppress inflammation by reducing TLR4/NF-κB pathway activation [31,67]. Depending on its qualitative and quantitative composition, the gut microbiota can either promote or prevent inflammatory states through its interactions with the immune system [67]. For example, lipopolysaccharides (LPS) present in the cell walls of Gram-negative bacteria induce acute inflammatory responses in the host [68]. Under normal conditions, the intestinal barrier, comprising the epithelial and mucosal layers, limits the translocation of LPS from the gut into the systemic blood circulation; however, increased gut permeability can compromise this barrier [67]. Elevated intestinal permeability facilitates the translocation of bacterial metabolites and their interaction with gut-associated lymphoid tissue (GALT), promoting low-grade systemic chronic inflammation [69]. Disruption of barrier integrity can lead to an increased influx of microbial substrates, mainly LPS, other inflammatory mediators, and cytokines, into the bloodstream [69]. Polyphenols exhibit prebiotic effects, as demonstrated in numerous studies conducted in animal models and humans [31]. They beneficially modulate the composition of the gut microbiota, mitigating and potentially preventing dysbiosis.

**Figure 3:**
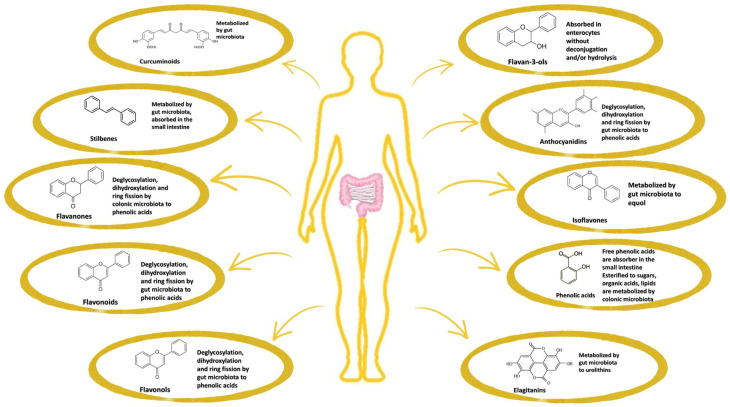
Transformation of phenolic compounds under the influence of gut microbiota.

In herbs, polyphenols are present as glycosides and complex oligomeric structures. They exhibit low bioavailability in the small intestine, with most of their metabolism occurring in the intestinal lumen via microbial activity [70]. Consequently, the gut microbiota is crucial for polyphenol absorption. Different bacterial taxa display affinities for distinct polyphenol groups. *Firmicutes* and *Bacteroidetes* are the primary colonic bacteria responsible for polyphenol metabolism, whereas *Flavonifractor plautii*, *Slackia equolifaciens*, *Slackia isoflavoniconvertens*, *Adlercreutzia equolifaciens*, *Eubacterium ramulus*, *Eggerthella lenta*, *Lactobacillus*, and *Bifidobacterium* spp. also play significant roles [31]. In the distal intestine, polyphenols undergo hydrolysis and metabolism, mediated by intestinal enzymes and gut microbiota (Figure 3). The resulting compounds are transported via portal circulation to the liver, where they undergo further biotransformation, followed by absorption through glucuronidation, sulfation, and catechol-O-methyltransferase-mediated pathways [71]. The interaction between the microbiota and polyphenols is bidirectional. On the one hand, the hydrolysis of glycosidic bonds in polyphenols produces glycans, which serve as essential nutrients for the gut microbiota, particularly for probiotic bacteria such as *Bacteroidetes* and *Lactobacillus*. In contrast, polyphenols contribute to alterations in microbiota composition, promoting the proliferation of SCFA-producing bacteria [31], which, in turn, prevents or reduces inflammation.

### 3.6. The Anti-Inflammatory Effect of Spice Herbs–Review of Studies

Spice herbs provide more than just flavour; many of them have documented anti-inflammatory properties, primarily due to polyphenols, terpenes, and other phytochemicals that modulate inflammatory pathways [47]. Spice herbs may play a key role as dietary anti-inflammatory agents, acting as PPAR activators, improving insulin sensitivity, counteracting dyslipidaemia, and reducing visceral fat accumulation [72]. These herbs inhibit NF-κB activation and enhance the expression of anti-inflammatory cytokines owing to their bioactive constituents [72]. Regular consumption may counteract the effects of chronic inflammation and consequently slow the progression of inflammation-related diseases. In a three-period, randomised, controlled crossover clinical trial, the effect of adding a blend of spices to a high-fat, high-carbohydrate meal was assessed in 12 overweight or obese men. Three meal variants were compared: without spices, with 2 g of the spice blend, and with 6 g of the spice blend (including oregano, coriander, basil, rosemary, and thyme). The results showed that consumption of the meal with 6 g of the spice blend significantly reduced the secretion of pro-inflammatory cytokines (IL-1β, IL-8, TNF-α) in peripheral blood mononuclear cells compared with the meal without spices. This effect was dose-dependent (6 g vs. 2 g), suggesting that spice herbs may modulate postprandial inflammatory responses in overweight or obese individuals. Although the effect of a single herb could not be isolated, the findings indicate a potential role of these herbs in attenuating acute inflammatory responses to a high-fat, high-carbohydrate meal, which may be relevant to metabolic inflammation and chronic disease risk [73]. In another three-period, randomised, crossover dietary trial involving overweight or obese adults with ≥1 cardiometabolic risk factor (*n* = 63), a four-week diet containing moderate to high doses of a spice blend (≈3.3–6.6 g/day), including herbs such as coriander, rosemary, oregano, basil, thyme, and sage, reduced the level of pro-inflammatory cytokines (fasting IL-6, postprandial IL-1β, IL-8, and TNF-α) and modulated the monocyte function. These results suggest that the regular inclusion of spice herbs in the diet may alleviate chronic low-grade inflammation associated with cardiometabolic risk [74].

Sage (*Salvia officinalis* L.) infusion administered to obese rats reduced their body weight, visceral fat, and serum CRP [75]. In mice with diet-induced obesity, a five-week course of ethanolic sage extract reduced inflammation (increased anti-inflammatory cytokines IL-2, IL-4, and IL-10 and decreased pro-inflammatory cytokines IL-12, TNF-α, and Keratinocyte Chemoattractant/Growth-Regulated Oncogene (KC/Gro)) in the plasma and inhibited adipocyte lipogenesis [76]. The anti-inflammatory properties of sage extracts have also been demonstrated in studies on isolated mature human subcutaneous adipocytes [77]. In RAW264.7 murine macrophages stimulated by LPS, sage extracts reduced the expression of pro-inflammatory cytokines TNF-α, IL-6, IL-1β, NF-κB, and nitric oxide levels, which play a key role in the inflammatory process [78]. NF-κB signalling plays a vital role in maintaining inflammation by promoting the transcription of various pro-inflammatory cytokines. Ethanolic *Salvia officinalis* extract improved inflammatory markers (TNF-α and prostaglandin E2) in carrageenan-induced inflammation in Wistar rats [79]. In mice with *Trichinella spiralis*-induced inflammation, sage extract effectively suppressed expression of inflammatory and fibrotic genes: FN1, TNF-α, TGF-β, and IL-10 [80]. Rosmarinic acid, a flavonoid present in sage, exhibited anticarcinogenic properties in murine models, possibly by inhibiting the MAPK pathway, suppressing ROS and NF-κB, and reducing the expression of the pro-inflammatory cyclooxygenase-2 gene [45].

Coriander (*Coriandrum sativum* L.) seeds and shoots are rich in essential oils and polyphenols, which have demonstrated hypoglycaemic and hypolipidaemic effects in obese and diabetic patients [50,81]. In contrast, diabetic patients receiving powdered coriander seeds showed marked improvements in metabolic syndrome and atherosclerosis indices, as well as increased cardioprotective markers [81]. In carrageenan-induced pleurisy in mice, hydro-alcoholic extracts of dried coriander leaves significantly reduced lung oedema, confirming their anti-inflammatory effects [82]. According to the cited authors, this effect was attributed to the presence of carotenoids, linalool (82.2%), pinene (4%), camphor (2.6%), δ-terpinene (2.6%), linalyl acetate (2.4%), and *p*-cymene (1.6%) in the leaves. However, in the cited study, no effect was observed on the number of leukocytes migrating into the pleural cavity of rats. The anti-inflammatory effect of methanolic coriander extract was demonstrated in a study conducted on female Wistar rats with ischaemia–reperfusion-induced liver injury [83]. In this study, rats receiving coriander extract showed a significant decrease in the expression of TNF-α, NF-κB, and caspase-3. Comparable results were observed in mice that received a polyphenol mixture isolated from coriander seeds [84].

The anti-inflammatory properties of Mexican oregano (*Lippia graveolens*, *Lippia palmeri*, and *Hedeoma patens*) were assessed using a murine cell model [85]. Phenolic extracts from the shoots of Mexican oregano exhibited anti-inflammatory effects: they significantly reduced the release of MCP-1 and IL-6, increased the secretion of IL-10, while not significantly reducing IL-1β expression. They also demonstrated the ability to scavenge the DPPH radical (2,2-diphenyl-1-picrylhydrazyl), although the potency of these effects depended on the plant variety used. In a study conducted on Hyla rabbits, supplementation with oregano essential oil (0.02%, 0.04% or 0.08%) led to a significant increasing the secretion of IL-2 and IL-10, and improved intestinal barrier [86]. The main constituents of oregano essential oil are carvacrol, linalool, *p*-cymene, γ-terpinene, and (E)-caryophyllene [87]. These compounds exhibit anti-inflammatory effects by attenuating lipopolysaccharide-induced IL-8 gene expression in BEAS-2B cells [87].

Basil (*Ocimum basilicum* L.) exhibits anti-inflammatory properties by modulating inflammatory mediators, including IL-10, IL-4, TNF-α, IFN-γ, and nitric oxide (NO) [88]. In asthmatic rats, aqueous-ethanolic *O. basilicum* extract (0.75, 1.50, and 3.00 mg/mL) increased IL-4 levels and decreased the IFN-γ/IL-4 ratio [89]. The anti-inflammatory properties of basil extract were demonstrated in a study on rats with ethanol-induced liver injuries. [90]. In this study, administration of basil extract at a concentration of 100 mg/kg resulted in a reduction of TNF-α, IL-6, and IL-1β levels compared to the control group. In a study using human leukocytes, basil extract did not reduce TNF-α and IL-6 levels; however, it significantly increased the level of the anti-inflammatory cytokine IL-10 [91]. The anti-inflammatory effects of basil extracts have also been observed in 3T3-L1 adipocytes co-cultured with RAW264.7 macrophages [92]. In this study, a decrease in the mRNA expression of the pro-inflammatory cytokines IL-6, IL-1β, TNF-α, NF-κB, and CCL2 (C-C motif chemokine ligand 2) was observed. The authors suggested that the anti-inflammatory action of basil extract in the context of adipocyte-induced inflammation may be mediated through the inhibition of Tnfrsf9 (TNF Receptor Superfamily Member 9) expression, a membrane receptor present on the surface of activated T lymphocytes, NK cells, monocytes, and dendritic cells.

The anti-inflammatory effects of thyme essential oil and some of its main constituents have been demonstrated in studies conducted in mice and cell lines, including THP-1 cells (a human acute monocytic leukaemia cell line), J774A.1, RAW 264.7 cells (murine macrophage cell lines), and human polymorphonuclear neutrophils [93]. Measurements of pro-inflammatory cytokine mRNA expression and secreted protein levels in LPS-activated THP-1 macrophages showed that thyme essential oil is a potent inhibitor of IL-6, IL-8, IL-1β, and TNF-α synthesis [94]. In a study on LPS-activated murine BV-2 microglial cells, thyme essential oil reduced the mRNA expression of IL-6 and TNF-α compared to that in control cells by modulating the NF-κB and C/EBPβ signalling pathways [93]. In another study, oil derived from *Thymus albicans* inhibited nitrite production in LPS-stimulated RAW 264.7 murine macrophages, a process catalysed by the pro-inflammatory inducible nitric oxide synthase [95]. Touaibia [96] demonstrated the anti-inflammatory effects of essential oils from *Thymus algeriensis* and *Thymus dreatensis* using erythrocyte membrane stabilisation assays in murine red blood cells and the heat-induced albumin denaturation method. In a study conducted in BALB/c mice with acne induced by *Cutibacterium acnes*, reduced NF-κB levels were observed in the ear tissue, approaching those of the control group [97].

The anti-inflammatory and antioxidant properties of rosemary are attributed to compounds such as carnosol, carnosic acid, rosmarinic acid, ursolic acid, oleanolic acid, hesperidin, and micromeric acids [98,99]. In a randomised, double-blind clinical trial, the effect of rosemary (*Rosmarinus officinalis* L.) supplementation on inflammatory markers in patients with rheumatoid arthritis was assessed. The study included 72 adults who received 4 g of powdered rosemary leaves or a placebo daily for 12 weeks while continuing their standard treatment. The intervention group showed significant reductions in CRP and erythrocyte sedimentation rate (ESR) compared to the placebo group, as well as improvements in clinical indicators of disease activity. These findings suggest that rosemary may exert anti-inflammatory effects in humans, most likely due to the presence of bioactive compounds (including carnosic acid, carnosol, and rosmarinic acid) which modulate key inflammatory pathways [100].

In the traditional medicine of many countries, various mint cultivars (fresh and dried) are used to help reduce body mass and for their anti-inflammatory, antioxidative, antidiabetic, and cardioprotective properties [101,102]. The active compounds include flavonoids (catechin, epicatechin, rutin, myricetin, luteolin, apigenin, naringenin, kaempferol, and quercetin) and phenolic acids (rosmarinic, gallic, chlorogenic, and caffeic) [103]. Peppermint may inhibit inflammation through activation of the AMPK/unc-51-like kinase 1/nuclear factor erythroid 2-related factor 2 (AMPK/ULK1/Nrf2) autophagy pathway, while simultaneously inhibiting NF-κB and mitogen-activated protein kinase signalling pathways, thereby suppressing the production of pro-inflammatory mediators and nitric oxide, and inducing the production of anti-inflammatory prostaglandins [104]. Peppermint essential oil (0, 25, 50, 100, and 200 µg/mL) significantly inhibited the secretion of IL-1β and TNF-α in LPS-stimulated porcine alveolar macrophages (PAMs) and demonstrated strong antioxidant activity in DPPH assays [105]. Comparable results were reported by Brahmi et al. [106] in a study using LPS-stimulated murine RAW 264.7 macrophages. In that study, ethanolic extracts of mint (*Mentha spicata* L., *Mentha pulegium* L., and *Mentha rotundifolia* L.) markedly reduced IL-6 secretion, while extracts of *Mentha pulegium* L. and *Mentha rotundifolia* L. additionally reduced MCP-1 and TNF-α secretion. A hydroethanolic extract of *Mentha pulegium* L. (10, 30, and 90 μg/mL) reduced the expression and biosynthesis of pro-inflammatory mediators TLR-4 and NF-κB in human peripheral blood mononuclear cells [107]. Other studies have also demonstrated the strong anti-inflammatory effects of extracts from various mint species [108,109,110].

Studies referred to in the articles included in our review on the effects of spice herbs on inflammation were conducted on humans (*n* = 5; Table 1), laboratory animals (*n* = 6; Table 2), and in vitro (*n* = 8; Table 3). The small number of available human studies is due to the specific nature of interventional studies (animals are more readily available, homogeneous, and can be maintained under identical environmental conditions with a defined diet, which minimises the influence of confounding factors and ensures reliable results). In contrast, human studies are more challenging to conduct due to heterogeneity, making it difficult to draw definitive conclusions. It is evident that the results of studies on animals cannot be fully extrapolated to humans; however, some of them (e.g., rats) are characterised by metabolic pathways similar to humans [111], suggesting a high likelihood that comparable results could be obtained in humans. Nevertheless, this represents a significant limitation that complicates interpretation.

## 4. Potential Spice Herb–Drug Interactions and Dosage

Comorbidities associated with metabolic syndrome (obesity, hyperglycaemia, hypertension, hyperlipidaemia, insulin resistance) require long-term pharmacological treatment. Some commonly used herbs may interact with medications, which can involve pharmacokinetic or pharmacodynamic interactions [112]. Pharmacokinetic interactions are primarily mediated by hepatic cytochrome P450 (CYP450) isoenzymes (CYP450) [112]. Certain bioactive compounds present in herbs, such as phenols, flavonoids, alkaloids, glycosides, coumarins, phytosterols, tannins, and saponins, may compromise CYP activity. As a result of absorption, distribution, metabolism, and excretion processes, drug concentrations in the body may be altered, potentially leading to reduced or enhanced effects of medication [113]. Pharmacodynamic interactions, on the other hand, may result in antagonistic, additive, or synergistic effects, which can be either beneficial or detrimental, depending on interactions between drugs and receptors in target organs [113]. Available studies on herb and drug interactions have been conducted in laboratory animals, which should be taken into account when interpreting the results. Laboratory animals, particularly rats, are always used in potentially hazardous experiments, and because their metabolic pathways are similar to those of humans, it can be assumed with a high likelihood that the results obtained will be comparable in humans [114]. Based on the review of studies on spice herbs included in our analysis, we found that:

- coriander–seeds of *Coriandrum sativum* increased the bioavailability of the hypoglycaemic drug Glimepiride (studies in rats) [115]. Coriander produced an additive effect and a moderate risk of hypoglycaemia when used with Metformin, Sitagliptin, and Gliclazide [116].
- sage-*Salvia miltiorrhiza* Bunge, in interaction with the anticoagulant Warfarin, inhibits CYP1A2, CYP2C9, CYP1E1, CYP2C6, and CYP2C11, while inducing CYP3A4 [117]. It increases steady-state plasma Warfarin concentrations in rats and reduces Warfarin clearance [118].
- mint-*Mentha piperita* interacts with drugs used in cardiovascular diseases, including inhibition of CYP3A4 (Felodipine), induction of CYP3A4 (Nifedipine), and induction of P-gp (Digoxin, Talinolol) [117].
- basil–in an in vivo study using human liver microsomes, aqueous and methanolic extracts inhibited CYP2B6, whereas ethanol and methanol extracts inhibited Rifampicin [118].

No information was found in the available literature regarding interactions between bioactive drug components and rosemary, oregano, or thyme.

When analysing interactions with drug components, the amount of active compounds capable of interacting is crucial. Therefore, to prevent adverse interactions, it seems safer to use these herbs only as spices, that is, in amounts not exceeding 1 g per day [119,120]. A review of studies on the health benefits of culinary doses of herbs and spices in the prevention and treatment of metabolic syndrome included black pepper, chilli, cardamom, cinnamon, coriander, cumin, fennel, fenugreek, garlic, ginger, Nigella seed, rosemary, sage, and turmeric [121]. In the cited studies, most dried herbs/spices were used in doses ranging from 1 to 6 g, which is representative of amounts typically used in cooking. These amounts were sufficient to exert beneficial effects on vascular function, postprandial blood glucose, insulin, and lipid levels. However, a study involving 120 individuals with metabolic syndrome recommended a therapeutic dose of 1 g of culinary spices (ginger, cinnamon, black seed) three times daily for 12 weeks [122]. In many other studies on type 2 diabetes, the therapeutic dose of spice herbs and spices (including black cumin, clove, parsley, saffron, thyme, ginger, black pepper, rosemary, turmeric, basil, oregano, and cinnamon) is set at a maximum of 3 g per day, although higher doses are sometimes used, e.g., 6 g of cinnamon. [123]. Considering the cited studies conducted in chronically ill individuals, this dose can be considered safe for people taking medications (the studies did not report drug discontinuation). A randomised, crossover, controlled dietary study involving 71 individuals at increased risk of cardiovascular disease, conducted in the USA, showed that daily consumption of a culinary spice blend at doses of 6.6 g, 3.3 g, or 0.5 g for 4 weeks reduced certain atherogenic parameters, with the 6.6 g dose being the most effective [124]. The blend contained six of the herbs analysed in our review: coriander, sage, basil, rosemary, oregano, and thyme. In the 6.6 g daily blend, coriander was present in the highest amount (0.833 g) and sage in the lowest (0.02 g). However, the study did not examine the effects of culinary herb consumption on drug efficacy. Due to variations in the doses of herbs used across different studies, it is difficult to draw definitive conclusions. Nevertheless, for the spice herbs analysed in our review, single-herb doses did not exceed 3 g and were considered safe.

## 5. Conclusions and Future Perspectives

Metabolic syndrome is a cluster of disorders that affects a substantial proportion of women, particularly during the perimenopausal period. The primary physiological hallmark of metabolic syndrome is chronic inflammation. The use of spice herbs as part of a nutritional strategy aimed at preventing chronic inflammation is supported by a growing body of scientific evidence. It should be emphasised that these studies are concerned with dietary support and prevention, rather than with treatments substituting standard medical therapy. Bioactive compounds present in spice herbs (e.g., polyphenols, terpenes, and essential oils) influence metabolic processes in the body primarily through several mechanisms: (1) inhibition of NF-κB, a key transcription factor responsible for the production of pro-inflammatory cytokines; herbal compounds may block activation of this pathway, thereby reducing the production of IL-6, TNF-α, and other inflammatory mediators; (2) activation of PPARs, nuclear receptors that regulate lipid and glucose metabolism and modulate cytokine activity; (3) prevention of gut dysbiosis, which improves intestinal barrier integrity and suppresses inflammation by reducing activation of the TLR4/NF-κB signalling pathway and preventing the translocation of bacterial metabolites and their interaction with GALT, processes that would otherwise promote systemic, chronic low-grade inflammation; and (4) antioxidant activity, neutralisation of free radicals, and reduction of oxidative stress, which is closely associated with chronic inflammation.

The recent trend of incorporating natural products into the prevention and supportive treatment of chronic diseases largely reflects patient concerns regarding the potential adverse effects of synthetic drugs. Traditional medicine is now approached from a modern scientific perspective. It focuses on elucidating the mechanisms of action of bioactive compounds present in food, enabling their targeted application. Importantly, these approaches are accessible and practical for patients, regardless of their nutritional, physiological, or medical knowledge. The incorporation of spice herbs into the daily diet may represent a simple and safe approach to increasing the intake of anti-inflammatory bioactive compounds. According to available literature, individual spice herbs are used in amounts not exceeding 3 g, which does not cause adverse effects on human health. Higher amounts, however, may affect the taste and aroma of dishes due to the content of active compounds, such as essential oils, which may be unacceptable to consumers.

Future research should focus on the precise determination of optimal doses and combinations of herbs to maximise benefits while avoiding potential adverse effects resulting from excessive intake of certain compounds or inappropriate selection of herbs. Active compounds may act synergistically, but they may also exert antagonistic effects. Future research on the use of spice herbs to reduce inflammation in metabolic syndrome should focus on elucidating these interactions. Analysis of the available literature indicates that existing studies are characterised by substantial variability in administered doses, forms of administration, duration of exposure, and the biological models used (cells, tissues, live animal models, and humans), which complicates the unequivocal interpretation of findings. Long-term studies conducted in larger populations of women with metabolic syndrome are required, as physiological differences, particularly those related to oestrogens, may result in sex-specific effects.

## Figures and Tables

**Table 1:** The effect of herbs on markers of inflammation in metabolic syndrome: A review of clinical studies.

Characteristic	Inflammation Parameters	Tissue	Experimental Factors	Type of Work	References
Patients with rheumatoid arthritis vs. Control (*n* = 72)	↓ TNF-α, ↓ IL-6	Serum	4 g/day (*Rosemary* leaf powder)	Double-blind randomized controlled trial	[100]
Overweight/obese men (*n* = 13) vs. Control	↓ Postprandial IL-1β, ↓ IL-6, ↓ TNF-α	Blood	6 g or 12 g per meal (Spice blend)	3-period, crossover, randomized controlled trial	[73]
Adults at risk of cardiometabolic disease (male *n* = 28, female *n* = 35)	↓ Plasma cytokines, altered monocyte function, ↓ IL-6	Plasma/Monocytes	2715–5785 g/day per meal of spice blend for 4 weeks	Randomized controlled trial, secondary outcome analysis	[74]
Type 2 diabetic patients (*n* = 40) vs. Control	↓ CRP (indirect)	Serum	1000 mg/day (*Coriander* seed powder)	Clinical trial	[81]
Adolescent girls with MetS (*n* = 70) vs. Control	↓ hs-CRP, ↓ IL-6	Serum	Mediterranean diet (high supply of polyphenols from herbs and olive oil)	Randomized trial	[39]

↓—decrease; hs-CRP—high sensitivity; IL-6—interleukine-6; TNF-α—tumor necrosis factor, CRP—*C*-reactive protein; MetS—metabolic syndrome; IL-1β—interleukin 1β.

**Table 2:** The effect of herbs on markers of inflammation in metabolic syndrome: A review of animal studies.

Animal Species		Inflammation Parameters	Place of Collection	Duration of Intervention	Experimental Factors	References
Male Wistar rats	*n* = 32	↓ IL-4, ↓ IFN-γ,	Serum/BALF	28 days	*Ocimum basilicum* hydro-ethanolic extract, orally, 100, 200, 400 mg/kg	[89]
Male Mice C57BL/6J	*n* = 48	↓ TNF-α, ↓ IL-6,	Serum/Adipose tissue	8 weeks	*Salvia officinalis* ethanolic extract, orally, 100, 300, 500 mg/kg	[76]
Rats Wistar	*n* = 36	↓ IL-1β, ↓ TNF-α, ↓	Serum/Liver	14 days	*Ocimum basilicum* aqueous extract, oral, 200, 400 mg/kg	[90]
Male Wistar rats	*n* = 42	↓ inflammatory markers	Paw tissue/Serum	7 days	*Coriandrum sativum* extract, orally, 100, 200, 400 mg/kg	[82]
Wistar rats	*n* = 40	↓ Apoptosis, ↓ inflammatory infiltration	Liver tissue	3 days	*Coriandrum sativum* extract, orally, 300 mg/kg	[83]
Growing Hyla rabbits	*n* = 192	↑ IL-2; ↑ IL-10	Duodenum, jejunum	35 days	Oregano essential oil, orally, 0.02%, 0.04%, 0.08%	[86]

↑—increase; ↓—decrease; IFN-γ—interferon gamma; TNF-α—tumor necrosis factor; CRP—*C*-reactive protein; IL-1β—interleukin 1β; IL-2—interleukin 2; IL-4—interleukin 4; IL-6—interleukin 6; IL-10—interleukin 10; BALF—Bronchoalveolar lavage fluid.

**Table 3:** The effect of herbs on markers of inflammation in metabolic syndrome: A review of in vitro studies.

Cells or Tissues	Inflammation Parameters	Place of Collection	Duration of Intervention	Experimental Factors	References
Human adipocytes	↓ IL-6, ↓ MCP-1	Cell supernatant	24–48 h	*Salvia officinalis* extract, in medium, 10, 25, 50 μg/mL	[77]
Murine RAW 264.7 acrophages	↓ Pro-inflammatory cytokines	Cell culture	24 h	*Mint extracts* ethanolic extract (25, 50, 100 μg/mL)	[106]
Adipocytes (3T3-L1) and macrophages	↓ IL-6, ↓ MCP-1	Co-culture medium	24 h	*Ocimum basilicum*	[92]
LPS-activated macrophages	↓ IL-6, ↓ TNF-α	Cell supernatant	24 h	*Thymus vulgaris* essential oil	[93]
Intestinal barrier model	↓ Inflammation	Caco-2 cells	24 h	*Mentha* spp. oil	[105]
Human PBMCs (Peripheral blood) (n = 10)	↓ TLR-4, ↓ NF-κB	Blood cells	24, 48, 72 h	*Mentha pulegium* L. extract (10, 20, 40, 80, 160, 320 μg/mL)	[107]
Human leukocytes	↑ IL-10	Cell culture	72 h	*Ocimum basilicum* rosmarinic acid extract	[91]
Human bronchial cells	↓ Pro-inflammatory cytokines	Lung cells	24 h	*Origanum minutiflorum* essential oil	[87]

↑—increase; ↓—decrease; MCP-1—Monocyte Chemoattractant Protein-1; TNF-α—tumor necrosis factor; IL-6—interleukine-6; IL-10—interleukin 10; NF-κB—Nuclear Factor Kappa B; TLR-4—Toll-like receptor 4.

## Data Availability

The original contributions presented in this study are included in the article. Further inquiries can be directed to the corresponding author.

## Abbreviations

The following abbreviations are used in this manuscript:

hs-CRP	High-Sensitivity *C*-reactive Protein
CRP	*C*-reactive Protein
IL-1, 1β, 4, 6, 8	Interleukin-1, 1β, 4, 6, 8
TNF-α	Tumour Necrosis Factor-alpha
NF-κB	Nuclear Factor kappa B
IFN-γ	Interferon gamma
BMI	Body Mass Index
PPAR-γ	Peroxisome Proliferator-Activated Receptor gamma
TLR4	Toll-like Receptor 4
ROS	Reactive Oxygen Species
NO	Nitric Oxide
HOMA-IR	Homeostatic Model Assessment for Insulin Resistance
NGF	Nerve Growth Factor
SII	Systemic Immune-Inflammation Index
SIRI	Systemic Inflammatory Response Index
DII	Dietary Inflammatory Index
PUFA	Polyunsaturated Fatty Acids
MUFA	Monounsaturated Fatty Acids
SFA	Saturated Fatty Acids
VEGF	Vascular Endothelial Growth Factor
EGF	Epidermal Growth Factor
SCFAs	Short-chain Fatty Acids
GM-CSF	Granulocyte–macrophage Colony-stimulating Factor
AMPK	AMP-activated Protein Kinase
MCP-1	Monocyte Chemotactic Protein-1
PAI-1	Type 1 Plasminogen Activator Inhibitor
C/EBPβ/α	CCAAT/enhancer-binding protein beta/alpha
LPS	Lipopolysaccharides
GALT	Gut-associated Lymphoid Tissue
KC/Gro	Keratinocyte Chemoattractant/Growth-Regulated Oncogene
CCL2	C-C Motif Chemokine Ligand 2
Tnfrsf9	TNF Receptor Superfamily Member 9
ESR	Erythrocyte Sedimentation Rate
AMPK/ULK1/Nrf2	AMPK/unc-51-like kinase 1/nuclear factor erythroid 2-related factor 2
PAMs	Porcine Alveolar Macrophages
